# Human Allergy to Cats and Dogs: Molecular and Environmental Determinants, Diagnosis, and Precision Management

**DOI:** 10.3390/vetsci13090905

**Published:** 2026-09-03

**Authors:** Jungwhan Chon, Kun-Ho Seo, Yeonghun Jee, Kihoon Kim, Kwang-Young Song

**Affiliations:** 1 Department of Food Science and Biotechnology, Kangwon National University, Chuncheon-si 24341, Republic of Korea; 1000cda@kangwon.ac.kr (J.C.);; 2 Center for One Health, College of Veterinary Medicine, Konkuk University, Seoul 05029, Republic of Korea; bracstu3@konkuk.ac.kr; 3 College of Veterinary Medicine, Kangwon National University, Chuncheon-si 24341, Republic of Korea; kihoon@kangwon.ac.kr; 4 Department of Companion Animal Health and Pet Industry, Daegu Haany University, Gyeongsan-si 38610, Republic of Korea

**Keywords:** pet allergy, cross-reactivity, asthma, rhinitis, immunotherapy

## Abstract

Cat- and dog-derived allergenic proteins are important indoor exposure sources, but allergen exposure, IgE sensitization, and clinically manifest allergy are distinct biological and clinical states. Major molecules include the cat secretoglobin Fel d 1 and several dog lipocalins, including Can f 1, together with serum albumins, kallikreins, and other components that can contribute to species-specific sensitization or cross-reactivity. These proteins are transported in the environment on heterogeneous carriers such as dander fragments, dust, and aerosol particles, allowing both direct and indirect exposure. Clinical relevance depends on the concordance among exposure, molecular sensitization, symptoms, and host factors rather than on environmental detection alone. This narrative review integrates molecular allergen biology with environmental dissemination, IgE-mediated immune mechanisms, clinical phenotypes, diagnostic interpretation, and management. Particular emphasis is placed on the distinction between molecular homology, IgE cross-reactivity, and clinically relevant cross-allergy; on the complementary role and limitations of component-resolved diagnosis; and on the variable evidence supporting environmental control and cat- versus dog-allergen immunotherapy. Standard pharmacotherapy remains central to symptom control, whereas biologics and allergen-neutralizing approaches are restricted to specific indications or remain investigational. A precision-management framework therefore requires molecular results to be interpreted in the context of exposure–response concordance, clinical phenotype, and inflammatory disease characteristics.

## Simple Summary

Cats and dogs release allergenic proteins from saliva, skin-associated secretions, dander, urine, and other biological materials. These proteins can be transported on airborne particles and dust, including into buildings without resident animals. Exposure alone, however, does not mean that a person will become sensitized or develop allergic disease. Clinical allergy depends on the interaction between the relevant allergen molecules, the amount and pattern of exposure, allergen-specific IgE, host susceptibility, and the affected organ system. This review summarizes the major cat and dog allergen molecules, how they are carried through indoor environments, the immune mechanisms that lead from sensitization to symptoms, and the strengths and limitations of current diagnostic and management approaches. Particular attention is given to component-resolved diagnosis, cross-reactivity, allergen immunotherapy, environmental control, and emerging allergen-neutralizing strategies. The review emphasizes that molecular sensitization results must be interpreted together with exposure history and clinical symptoms before a causal allergen is assigned.

## 1. Introduction

Cats and dogs are important sources of indoor allergenic proteins in modern living environments [1]. Their presence increases opportunities for exposure, but exposure itself is not equivalent to immunological sensitization, and sensitization does not necessarily result in symptomatic allergic disease. The clinical relevance of contact with animal allergens depends on the amount and timing of exposure, individual susceptibility, and the presence of allergen-specific IgE together with a compatible clinical history [2,3,4]. Urbanization, indoor lifestyles, and changes in patterns of human–animal contact may alter exposure opportunities, but these factors should not be interpreted as direct causes of increasing allergy prevalence [1].

Cat and dog allergens originate from several biological materials, including saliva, skin-associated secretions and dander, urine, serum, and fur contaminated with secreted proteins [2,3,4]. From a molecular perspective, Fel d 1, a secretoglobin, is the dominant cat allergen, whereas dog allergy is molecularly more heterogeneous and commonly involves lipocalins such as Can f 1 as well as other components [2,5]. Allergen molecules should be distinguished from the environmental particles that carry them: proteins such as Fel d 1 and Can f 1 can associate with dander fragments, dust, and aerosol particles spanning a broad size range. Consequently, deposition in the respiratory tract depends on the aerodynamic properties of the carrier particle and inhalation conditions rather than on a fixed “allergen particle size” [3,4].

Pet ownership and close human–animal contact are common in many populations, and cat and dog allergens are frequently detected in homes, schools, workplaces, and public environments, including locations without resident animals [6,7]. These observations demonstrate widespread exposure but do not by themselves establish a causal increase in allergic disease. Sensitization frequencies vary according to population, age, geography, host atopy, and exposure patterns; therefore, environmental allergen levels and pet ownership should be interpreted as context-dependent contributors rather than universal determinants of sensitization [2,3,4].

In susceptible individuals, clinically relevant cat or dog allergy is most often mediated by allergen-specific IgE and type I hypersensitivity, with manifestations that include allergic rhinitis, asthma, and conjunctivitis and, in some patients, atopic dermatitis [2,3]. The pathway from exposure to disease is not deterministic. Host susceptibility, genetic predisposition, age and timing of exposure, environmental and microbial context, and the intensity and pattern of allergen exposure can influence whether exposure is followed by sensitization or clinically apparent allergic disease [2]. This distinction is essential when interpreting environmental measurements and molecular diagnostic results.

**Figure 1:**
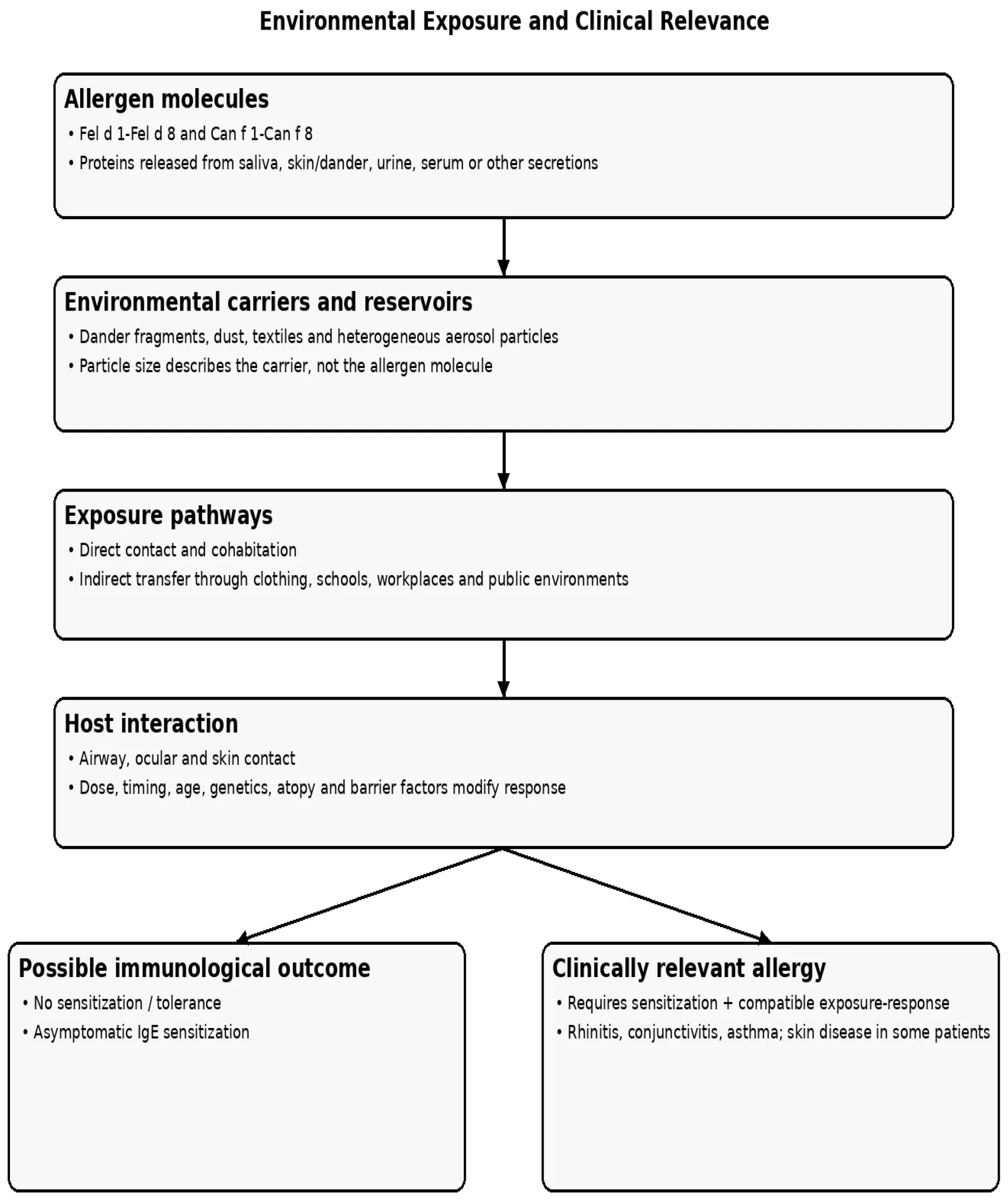
Environmental pathway linking cat and dog allergen molecules, heterogeneous carrier particles and reservoirs, exposure routes, and clinical relevance. The figure emphasizes that exposure, IgE sensitization, and clinically relevant allergy are distinct states. Created by the authors based on information from references [2,3,4].

Recent reviews have separately emphasized diagnostic challenges in dog and cat allergy [4], component-resolved diagnostics [8], broad allergen sources and pathogenesis [1], and the favorable and unfavorable consequences of exposure to domestic and non-domestic animals [9]. The present review extends these perspectives by explicitly integrating four levels that are often discussed separately: (i) WHO/IUIS-defined allergen molecules, (ii) the environmental carriers and exposure conditions through which these molecules are encountered, (iii) the immunological transition from exposure to sensitization and clinical disease, and (iv) the interpretation of molecular diagnostics in relation to clinical phenotype and management. Throughout the review, exposure, IgE sensitization, and clinically relevant allergy are treated as distinct concepts, and molecular homology is distinguished from demonstrated IgE cross-reactivity and from clinically relevant cross-allergy. The integrated pathway linking allergen sources, environmental carriers, exposure routes, IgE sensitization, and clinically relevant allergy is summarized in Figure 1.

## 2. Methods

This article was prepared as a narrative review. Literature relevant to human allergy to cats and dogs was identified through PubMed/MEDLINE and by screening the reference lists of relevant reviews and selected articles, with publications considered from database inception through July 2026. Search concepts combined terms related to cat, dog, companion animal, Fel d, Can f, allergen exposure, sensitization, molecular allergology, component-resolved diagnosis, cross-reactivity, asthma, rhinitis, immunotherapy, environmental control, and allergen neutralization. English-language, peer-reviewed human studies, consensus documents, systematic or narrative reviews, and mechanistic studies directly relevant to the interpretation of human allergy to cats or dogs were considered. Primary studies were preferentially used to support quantitative estimates of exposure, sensitization, diagnostic findings, and clinical effects, whereas reviews and consensus documents were used primarily for evidence synthesis and established terminology. Studies were selected according to their relevance to the predefined scope and review questions rather than through a formal systematic-review or meta-analytic protocol. Critical appraisal focused on the study population, exposure or sampling matrix, allergen assay, molecular component evaluated, definition of clinical outcomes, methodological limitations, and whether the reported observation concerned environmental exposure, immunological sensitization, or clinically relevant allergy. Because this was a narrative review, no pooled effect estimates were calculated.

## 3. Origin and Characteristics of Allergens

### 3.1. Origin and Distribution of Allergens

Allergenic proteins from cats and dogs are released from saliva, skin-associated secretions and dander, urine, serum, and other tissues or secretions, and may subsequently contaminate fur and indoor reservoirs [9,10,11]. Grooming can distribute salivary proteins over fur and skin, after which allergen-containing biological material may dry, fragment, and become incorporated into dust or airborne particles [4]. The amount and route of exposure therefore depend on both animal-derived secretion patterns and the physical behavior of the environmental carrier.

Allergen molecules are proteins or glycoproteins and should not be assigned a single aerodynamic diameter. In indoor environments, they are associated with heterogeneous carriers, including dander fragments, dust particles, fibers, and aerosol droplets. The probability of deposition in the nose, conducting airways, or more distal respiratory tract depends on the aerodynamic properties of the carrier particle, breathing pattern, and other deposition mechanisms; therefore, allergen-containing particles should not be assumed to uniformly reach the alveolar region [3,4].

### 3.2. WHO/IUIS-Recognized Cat and Dog Allergens

The WHO/IUIS Allergen Nomenclature database recognizes Fel d 1-Fel d 8 for domestic cat and Can f 1-Can f 8 for domestic dog. Table 1 therefore lists all currently recognized components rather than only components available on selected diagnostic platforms. Reported sensitization frequencies are cohort- and assay-dependent; where robust component-specific estimates are not established or the component is not routinely available in clinical assays, this is stated explicitly. “Major allergen” is used only when supported by a high prevalence of IgE recognition in an appropriately defined allergic population, and membership in a protein family is not treated as evidence of clinical cross-reactivity.

### 3.3. Cat (Felis Domesticus)

Among cat allergens, Fel d 1 is the major allergen and a principal target of IgE responses in patients with cat allergy [25]. Fel d 1 is a protein belonging to the secretoglobin family and is primarily produced in sebaceous, salivary, and anal glands, from which it is released into the environment through fur and skin [4]. This protein is highly persistent in indoor environments and can induce allergic reactions even at very low concentrations [26,27].

Fel d 2 belongs to the serum albumin family and is found in blood, saliva, and skin. As this protein shares high structural similarity with albumins from other animal species, it serves as a major cause of cross-reactivity [3]. For example, the “pork-cat syndrome,” in which patients with cat allergy react to pork, is explained by cross-reactivity between Fel d 2 and porcine albumin [4].

Fel d 4 and Fel d 7 belong to the lipocalin family, originate from body fluids, and are characterized by their ability to disperse readily into the air. Lipocalin proteins function in the transport of hydrophobic molecules and exhibit high structural conservation, raising the possibility of cross-reactivity across species [3,27].

### 3.4. Dog (Canis Familiaris)

Dog allergens exhibit greater diversity than cat allergens, with multiple major allergens identified. Representative major allergens include Can f 1, Can f 2, Can f 4, and Can f 6, all of which belong to the lipocalin protein family [2,3,4]. These proteins are primarily derived from salivary tissues and exhibit IgE-binding activity [5].

Can f 5 possesses unique characteristics among dog allergens, as it is a kallikrein-family enzyme produced in the prostate gland. This allergen is generated exclusively in male dogs and is released into the environment through urine [3]. Can f 5 exhibits structural similarity to human prostate-specific antigen, which provides a potential molecular basis for IgE cross-reactivity [3]. Can f 3 belongs to the serum albumin family and exhibits high homology with albumins from other mammals, which may provide a molecular basis for IgE cross-reactivity across species; however, molecular homology alone does not establish clinically relevant cross-reactivity [2,3,4].

These molecular characteristics have implications for allergy diagnosis and treatment. Conventional extract-based diagnostic tests are limited in their ability to distinguish cross-reactivity, whereas component-resolved diagnosis (CRD) can provide additional information for evaluating molecular sensitization patterns and potential cross-reactivity [2,3,4].

## 4. Immunological Mechanisms of Allergy

### 4.1. Process of Allergic Sensitization

Immunological responses to dog- and cat-derived allergens are primarily mediated through IgE-mediated type I hypersensitivity reactions, which proceed through a complex series of immunological events [28,29]. When allergens enter the body through the respiratory tract, they first interact with bronchial mucosa and epithelial cells. During this process, epithelial cells secrete alarmin cytokines, such as thymic stromal lymphopoietin, IL-25, and IL-33, thereby initiating immune responses [30]. Subsequently, allergens are captured and processed by antigen-presenting cells, including dendritic cells, and are presented to naïve CD4+ T cells through MHC class II molecules [28]. A specific cytokine environment, particularly the presence of IL-4, promotes the differentiation of T cells into the Th2 phenotype, which represents a critical step in allergic responses [31].

Activated Th2 cells produce cytokines with distinct functions. IL-4 and IL-13 are central signals promoting B-cell class switching toward IgE, whereas IL-5 primarily supports eosinophil differentiation, recruitment, and survival rather than directly inducing IgE class switching [28]. The generated allergen-specific IgE circulates through the bloodstream and binds to FcεRI (high-affinity IgE receptors) expressed on the surface of mast cells and basophils [31,32]. Upon re-exposure to the same allergen, the allergen cross-links FcεRI receptors bound to IgE, thereby inducing mast cell degranulation [28]. During this process, inflammatory mediators such as histamine, leukotrienes, and prostaglandins are released, resulting in vasodilation, increased mucus secretion, and bronchoconstriction, which manifest clinically as allergic rhinitis, asthma, and conjunctivitis [29].

**Figure 2:**
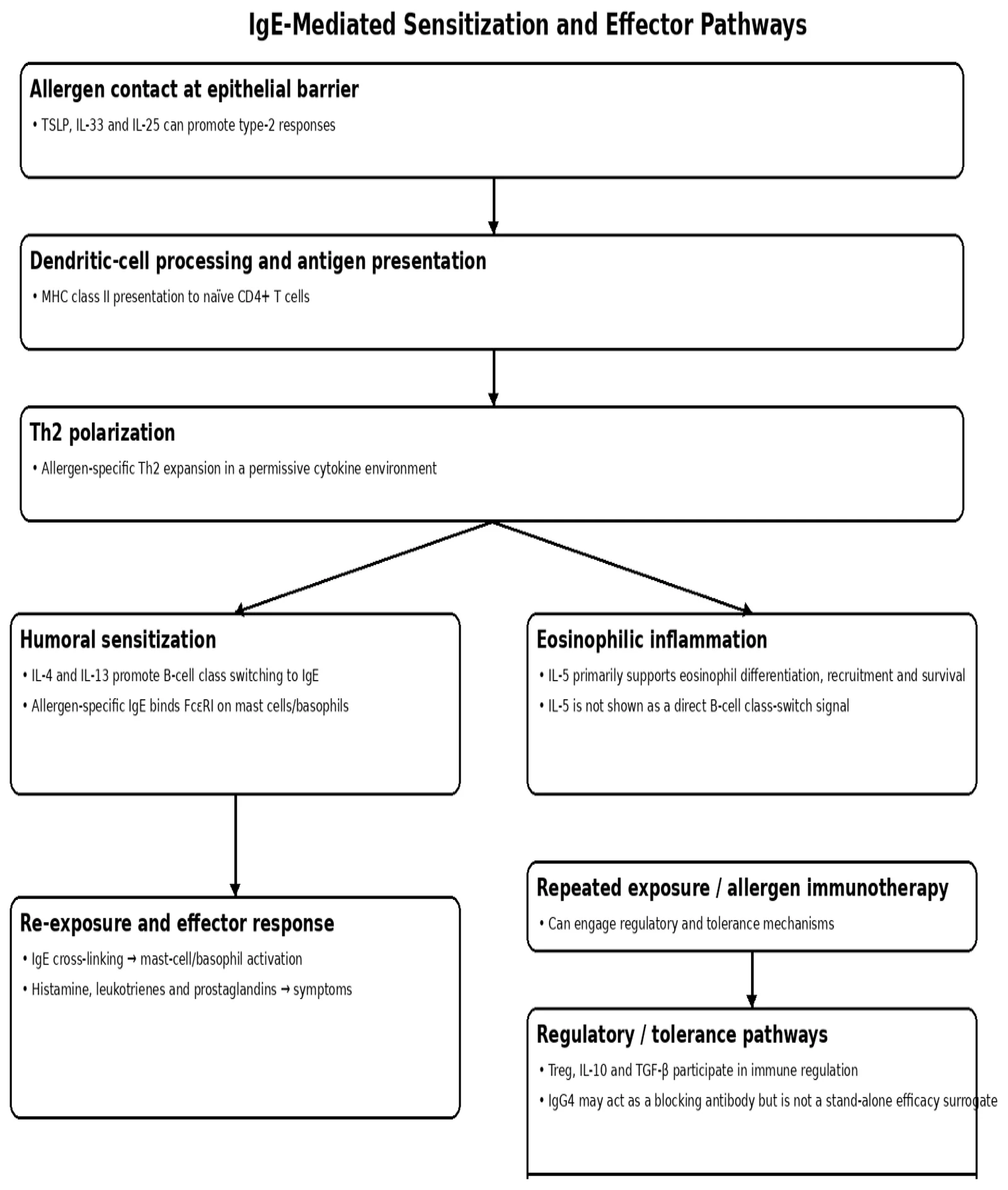
Immunological mechanisms of IgE-mediated allergic sensitization to cat and dog allergens. Created by the authors based on information from references [2,3,4].

Late and chronic responses may involve eosinophils, Th2 cells, ILC2 cells, and tissue-remodeling pathways [30]. Fel d 1 is the major cat allergen, whereas dog allergy is molecularly more heterogeneous and may involve multiple components, including Can f 1, Can f 2, Can f 3, Can f 4, Can f 5, and Can f 6 [2]. Accordingly, the immunological response to cat and dog allergens should be interpreted at the level of individual allergenic components rather than by attributing a uniform immunological property to all dog allergen components. The immunological sequence from epithelial allergen recognition to Th2 polarization, IgE production, mast-cell activation, and clinical allergic responses is illustrated in Figure 2.

### 4.2. Regulatory Role and Clinical Interpretation of IgG4

Although IgE antibodies play a central role in allergic immune responses, IgG4 antibodies perform immunoregulatory functions [33]. IgG4 is generally produced under conditions of repeated and long-term antigen exposure and is closely associated with immune tolerance [28,32]. During acute or initial allergen exposure, Th2 responses predominate, increasing IgE production and promoting allergic reactions. However, under persistent and low-dose repeated exposure, the immune system gradually develops regulatory responses, during which immunosuppressive cytokines, such as IL-10 and TGF-β, increase and promote IgG4 production [28,32].

Allergen-specific IgG4 can increase during repeated allergen exposure and during successful allergen immunotherapy and can function as a blocking antibody by competing with IgE-dependent allergen recognition [28,33]. However, IgG4 is one element of a broader regulatory response that includes Treg cells, IL-10, TGF-β, changes in effector-cell activation, and altered antibody quality. An increase in allergen-specific IgG4 is therefore best regarded as a mechanistic or pharmacodynamic marker rather than a routinely accepted stand-alone surrogate of individual clinical AIT efficacy. Clinical response must be assessed using symptoms, medication use, provocation outcomes where appropriate, and disease control [32].

### 4.3. Hygiene Hypothesis and Microbiome-Based Immune Regulation

A theory explaining the increasing prevalence of allergic diseases is the hygiene hypothesis, which proposes that microbial exposure during early life plays an important role in immune system development [34,35]. According to this hypothesis, exposure to diverse microorganisms and environmental antigens during childhood maintains the balance between Th1 and Th2 immune responses, thereby reducing the incidence of allergic diseases [35].

Companion animals may influence the microbial characteristics of indoor environments because pets are among the sources contributing to indoor microbial communities [36]. Indoor microbial exposure has been associated with allergic and inflammatory diseases, although the direction and magnitude of these associations vary across studies [36]. Such microbial exposures may influence allergic disease through multiple immunological mechanisms, including modulation of Th2-associated responses and regulatory immune pathways [35]. However, these effects are not consistently protective, and the relationship between microbial exposure and allergic disease appears to be context-dependent [35,36].

The diversity and composition of indoor microbial communities are influenced by multiple environmental and anthropogenic factors, including building characteristics, ventilation, occupancy, and occupant lifestyles [36]. Similarly, the relationship between companion animal exposure and allergic disease is not uniform. Early exposure to dogs has been associated with a reduced likelihood of sensitization in some studies, whereas findings for cat exposure have been inconsistent, and the timing and level of exposure may influence these outcomes [4]. Therefore, companion animal exposure should be understood within a broader environmental and immunological context rather than as uniformly protective or harmful.

## 5. Exposure and Transmission

### 5.1. Direct Exposure

Exposure may occur through direct physical contact with an animal or indirectly through allergen-containing material in a shared indoor environment. These exposure situations do not result in equivalent exposure levels, because actual exposure varies with the intensity and pattern of exposure and the characteristics of the indoor environment [4,37].

When companion animals are kept indoors, allergens can accumulate on textile surfaces, such as carpets, bedding, and sofas, which may function as allergen reservoirs [26]. These accumulated allergens may become airborne following environmental disturbance, resulting in intermittent or repeated inhalation exposure rather than a constant airborne concentration. Such repeated exposure may contribute to symptom elicitation in sensitized individuals, whereas the development of sensitization depends on multiple host- and exposure-related factors [3,4]. In addition, occupational groups with frequent animal contact, including veterinarians and animal-care workers, may experience substantial allergen exposure and an increased risk of work-related allergic symptoms [10].

### 5.2. Indirect Exposure

Indirect exposure refers to exposure pathways through which allergens are encountered even in environments where companion animals are not directly kept or contacted. Pet-derived allergens can be readily transported through various carriers such as human clothing, shoes, hair, and bags, and this kind of passive transfer contributes to widespread contamination of indoor environments [10,38]. Cat allergens have been found in school classrooms without cats at concentrations higher than those found in homes without cats but lower than those found in homes with cats [7]. For example, Fel d 1 and Can f 1 have been detected in school dust, including in environments where cats or dogs had no direct access, demonstrating the potential for indirect transfer of cat- and dog-derived allergens [39].

Pet-derived allergens have been detected in other public facilities such as public transportation systems, hospitals, hotels, and offices, potentially leading to persistent low-level exposure even among individuals without direct contact with companion animals [3]. Such indirect exposure may trigger symptoms particularly in sensitive individuals and represents a major factor complicating exposure avoidance strategies. Moreover, indirect exposure may also facilitate long-range transport through association with airborne fine particles [10]. This indicates that allergens are not confined to specific spaces but may exist across different environments.

### 5.3. Exposure Levels

Environmental studies have reported Fel d 1 and Can f 1 concentrations in settled house dust in relation to sensitization and asthma, but reported exposure levels should not be interpreted as universal biological thresholds. In a study of 12–14-year-old schoolchildren in Los Alamos, New Mexico, Fel d 1 and Can f 1 were measured in settled house dust using two-site immunoassays. Fel d 1 concentrations of ≥8 μg/g dust and Can f 1 concentrations of ≥10 μg/g dust were used to characterize relatively high household exposure; however, current household allergen concentrations were not clearly associated with sensitization or symptoms in individual children [40]. Thus, these values should be interpreted as study-specific exposure levels rather than universal clinical thresholds.

Exposure–response relationships vary according to the study population, individual susceptibility, exposure setting, sampling method, analytical approach, and clinical outcome. The persistence of detectable allergen in dust or on surfaces indicates environmental reservoirs, but detection alone does not establish clinically relevant exposure or symptomatic disease [37].

## 6. Cross-Reactivity and Polysensitization

### 6.1. Cross-Reactivity

Immunological cross-reactivity, in which proteins from different species are similarly recognized by IgE antibodies, frequently occurs across species [3,25]. Such cross-reactivity represents a major factor complicating the diagnosis and treatment of allergic diseases [41,42].

Cross-reactivity requires shared IgE-recognized epitopes and should be distinguished from simple membership in the same protein family. Structural homology can provide a molecular basis for cross-reactivity, but homology alone does not demonstrate IgE cross-reactivity, and IgE cross-reactivity does not necessarily translate into clinically relevant cross-allergy. These distinctions are particularly important for lipocalins and serum albumins [3,42].

A representative example is the cross-reactivity between the cat allergen Fel d 2 (serum albumin) and porcine Sus s 1 (serum albumin), which underlies pork-cat syndrome. IgE inhibition studies have shown that sensitization to cat serum albumin can represent the primary event, with subsequent cross-reactivity to porcine serum albumin and allergic reactions following pork consumption [13].

Several mammalian lipocalins show sequence and structural similarity. For example, IgE cross-reactivity between Fel d 4 and Can f 6 has been demonstrated in molecular studies [22,43]. In clinical interpretation, however, a positive IgE result to both components can reflect true co-sensitization, cross-reactive IgE, or both. Thus, IgE positivity should not be interpreted as a false-positive result solely because the original sensitizing source or clinical relevance remains uncertain.

Another example is the IgE cross-reactivity between the dog allergen Can f 5 and human prostate-specific antigen (PSA), both members of the kallikrein protein family. The clinical relevance of this cross-reactivity has been demonstrated in a case of human seminal plasma allergy [44]. This finding suggests that pet-derived allergens can also cross-react with homologous human proteins. Such diverse cross-reactivities complicate the identification of the true causative allergen, and conventional extract-based diagnostic methods are limited in their ability to distinguish these differences [2,4]. Therefore, CRD can help distinguish cross-reactivity from genuine sensitization [3].

**Figure 3:**
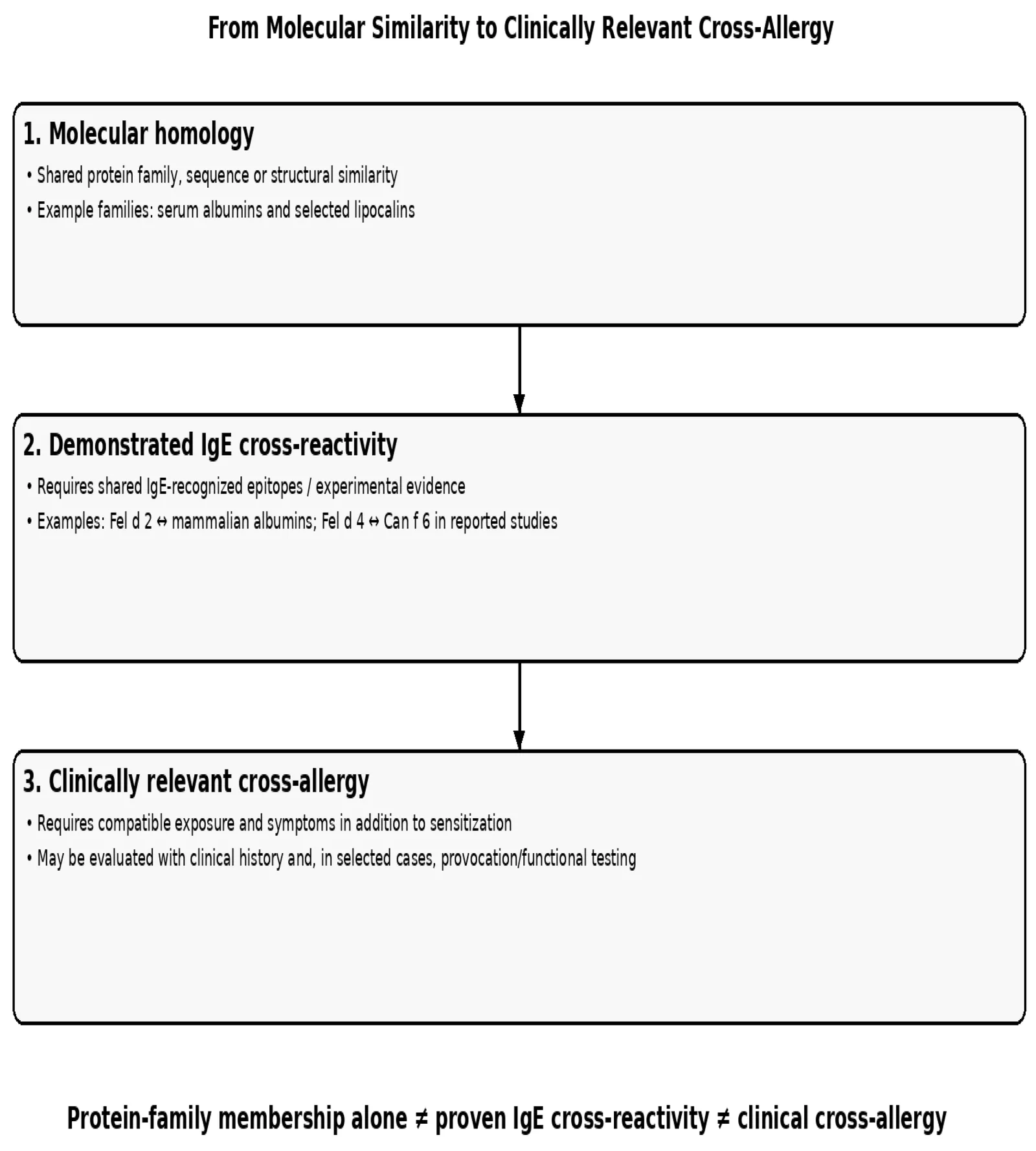
Conceptual hierarchy separating molecular homology, demonstrated IgE cross-reactivity, and clinically relevant cross-allergy. Representative examples are shown without implying that all members of a protein family are clinically cross-reactive.

Figure 3 distinguishes three levels of evidence in molecular allergology: protein-family or sequence homology, experimentally demonstrated IgE cross-reactivity, and clinically relevant cross-allergy supported by exposure–response concordance. This framework prevents structural similarity from being interpreted as proof of clinical cross-reactivity.

### 6.2. Polysensitization

Polysensitization refers to IgE sensitization to multiple allergen sources or molecular components and may reflect co-sensitization, cross-reactive IgE, or a combination of both [3]. Some cohorts have associated broader molecular sensitization profiles with asthma and greater disease severity; however, sensitization profiles alone do not establish clinical severity or relevance [3,8]. Interpretation should therefore consider the magnitude and molecular pattern of IgE sensitization together with exposure history, symptom timing, comorbidities, and clinical phenotype [3].

CRD can refine the characterization of a polysensitized patient by identifying the particular molecular components recognized by IgE. It may help distinguish primary sensitization from potential cross-reactivity, but it does not by itself establish which allergen causes symptoms. The results must therefore be integrated with exposure history and symptom timing and, when clinical relevance remains uncertain, with provocation testing or other functional assays [3,8].

Polysensitization may arise from complex interactions among exposure to multiple allergens, cross-reactivity, and immune responses. Therefore, evaluation and treatment of patients with animal allergies require approaches that consider the presence of sensitization as well as the underlying mechanisms and clinical significance of that sensitization.

## 7. Clinical Manifestations

### 7.1. Respiratory Diseases

Clinically relevant cat and dog allergy most commonly presents with allergic rhinitis, conjunctivitis, and/or asthma in sensitized individuals with a compatible exposure history [29]. These reactions occur after allergen-containing carrier particles or contaminated material reach the upper or lower airways or ocular mucosa; they should not be described as a consequence of a single fixed “allergen particle size” [3,4].

Allergic rhinitis is the most common clinical manifestation and is characterized by symptoms such as sneezing, rhinorrhea, nasal congestion, and nasal itching. These symptoms result from IgE-mediated inflammatory reactions and mast cell degranulation within the nasal mucosa and may progress to chronic inflammatory conditions with repeated exposure [45]. In particular, the major cat allergen Fel d 1 is readily airborne, with a substantial fraction carried on small particles that can remain suspended for prolonged periods [26].

Bronchial asthma represents a more severe respiratory disease characterized by airway inflammation, airway narrowing, excessive mucus secretion, and airway hyperresponsiveness [31]. In sensitized patients, repeated exposure can induce airway epithelial damage and infiltration of immune cells, which may lead to chronic inflammation and structural remodeling [30]. In genetically predisposed individuals, sensitization to pet-derived allergens has been associated with an increased risk of asthma development, whereas early-life exposure to companion animals may, under certain conditions, be associated with a reduced risk of subsequent allergic sensitization [4,46].

Exposure to cat allergens may induce both early and late asthmatic responses. In cat-allergic patients with asthma, late asthmatic responses following allergen challenge may occur up to 24 h after exposure [47].

Dog allergen sensitization has been associated with asthma severity in children. In particular, sensitization to Can f 2 and broader multisensitization to animal-derived lipocalins has been reported more frequently in children with severe asthma than in those with controlled asthma [48,49].

### 7.2. Cutaneous and Systemic Allergic Reactions

Dog- and cat-derived allergens may induce cutaneous and systemic reactions. A representative cutaneous manifestation is atopic dermatitis, which results from combined abnormalities in skin barrier function and immunological responses [50]. Atopic dermatitis is characterized by symptoms such as pruritus, erythema, xerosis, and lichenification, and may be triggered by inhalant allergens in susceptible individuals [50]. Exposure to allergens such as Fel d 1 may trigger an immune response [4]. Allergic conjunctivitis is also a common manifestation and is characterized by ocular itching, conjunctival redness, and increased tear secretion. This condition results from IgE-mediated reactions and mast cell activation within conjunctival tissues [29].

Systemic reactions attributable to cat or dog allergens appear to be uncommon compared with respiratory and ocular manifestations. Anaphylaxis represents a rare clinical manifestation of cat or dog allergy. When anaphylaxis occurs, it is potentially life-threatening and requires immediate treatment [51]. In addition, systemic reactions may occur in some patients through clinically relevant cross-reactivity between pet-derived and food allergens [13,42].

### 7.3. Factors Influencing Disease Severity and Clinical Heterogeneity

Clinical manifestations of cat and dog allergy show substantial heterogeneity among patients, reflecting the influence of multiple host-, exposure-, and allergen-related factors [3]. Sensitization to individual allergen components may be associated with distinct clinical phenotypes; for example, monosensitization to Can f 5 has been associated with selective clinical reactivity to male rather than female dogs [52]. However, associations between sensitization to individual allergen components or polysensitization and clinical severity should be interpreted probabilistically rather than deterministically. Neither sensitization to a single component, such as Fel d 1 or Can f 5, nor polysensitization alone should be regarded as a direct measure of symptom severity [3].

Genetic susceptibility, allergen exposure, and environmental factors such as air pollution can influence clinical manifestations and disease severity [30]. In particular, environmental exposures during infancy may influence subsequent allergic sensitization and the risk of allergic disease later in childhood [46]. For precision management, molecular sensitization profiles should be interpreted together with the patient’s clinical phenotype, inflammatory response pattern, and exposure history rather than used in isolation [3].

## 8. Diagnosis

### 8.1. Traditional Diagnostic Methods

Diagnosis of human allergy to cats or dogs requires concordance among a compatible clinical history, relevant exposure, and evidence of IgE sensitization [53]. Skin prick testing and serum allergen-specific IgE testing remain first-line tools for documenting sensitization, but a positive test does not by itself establish that the animal exposure is the cause of symptoms. Table 2 summarizes conventional and molecular approaches and emphasizes their complementary roles rather than ranking CRD as inherently more clinically specific.

Serum-specific IgE assays quantitatively measure IgE antibodies against specific allergens in patient blood samples and are used as alternatives when SPT is difficult to perform, such as in patients with skin diseases or those taking antihistamines [54]. Standardized methods such as ImmunoCAP provide high reproducibility and objectivity and enable quantitative comparison among various allergens [54].

Despite their widespread use, these conventional extract-based diagnostic methods have several limitations. First, allergen extracts consist of complex mixtures of proteins and therefore cannot distinguish individual allergen components, making it difficult to differentiate cross-reactivity from genuine sensitization [3]. For example, IgE responses to common proteins such as serum albumins may appear as cross-reactivity among different animal species, thereby obscuring the true sensitizing source [42]. Second, the composition of allergen extracts may vary substantially depending on manufacturing processes and source materials, resulting in limitations in standardization [2,3,4].

Third, results from SPT and specific IgE assays do not always correspond directly with clinical symptoms. In other words, sensitization may be present without clinical allergy [29]. This indicates that exposure history and symptom patterns must always be considered together during diagnosis. Furthermore, in the case of pet-derived allergens, many patients exhibit polysensitization patterns involving simultaneous reactions to multiple animals or allergens, making it difficult to identify the precise causative allergen using extract-based methods alone [2,3,4].

### 8.2. Molecular Diagnosis (CRD)

Component-resolved diagnosis (CRD) measures IgE to individual allergen molecules and can refine a sensitization profile beyond whole-extract testing [2,8]. It is particularly useful for identifying species-associated marker components and for recognizing patterns compatible with serum-albumin or lipocalin cross-reactivity. Nevertheless, CRD does not independently establish clinical causality, and its results should be interpreted together with the patient’s clinical history and relevant exposure information [42].

Associations between individual allergen components, polysensitization patterns, asthma, and disease severity have been reported; however, the role of allergenic molecules as predictors of disease severity requires further investigation [8]. CRD may therefore contribute to patient characterization and diagnostic clarification, but should not be used as a stand-alone predictor of disease severity or determinant of treatment.

When extract tests, component results, exposure history, and symptoms are discordant, additional evaluation may be considered in specialist practice. Controlled nasal allergen provocation can help establish the clinical relevance of sensitization in selected patients, while basophil activation testing may provide additional functional diagnostic information when conventional diagnostic approaches are inconclusive or conflicting [55,56]. These tests are not required routinely, but they illustrate the distinction between demonstrating sensitization and establishing clinically relevant allergy.

CRD is limited by the component repertoire of each platform, assay-specific performance, cost, accessibility, and the expertise required to interpret complex profiles. Accordingly, CRD should complement—not replace—clinical history, examination, and conventional allergy testing. Its contribution to personalized management is primarily to refine molecular sensitization profiles and patient classification, while treatment decisions should remain based on the overall clinical context rather than on CRD results alone [8].

## 9. Treatment and Management

### 9.1. Allergen Avoidance

Management begins by reducing clinically relevant exposure where feasible, but allergen avoidance should not be described as universally definitive or sufficient. The practical objective is to reduce exposure to an allergen that is both immunologically and clinically relevant, while recognizing the social and emotional consequences of pet removal. Because allergen reservoirs can persist after an animal is removed, environmental exposure may continue and additional environmental measures may still be required [26]. Decisions should be individualized according to disease severity, exposure–response concordance, and patient preferences. The principal management strategies for pet allergy, together with their advantages and limitations, are summarized in Table 3.

### 9.2. Environmental Control

Environmental control can reduce measured concentrations of airborne or reservoir allergens, but a reduction in allergen concentration does not necessarily produce an equivalent clinical improvement [37,57]. HEPA filtration, reservoir reduction, cleaning, and bedroom exclusion can be considered as parts of a multimodal strategy. Their clinical effectiveness varies with adherence, baseline exposure, room characteristics, and the dominant route of exposure; therefore, environmental allergen reduction and patient-centered clinical benefit should be reported as distinct outcomes.

Regular cleaning may help reduce allergen accumulation within the environment. In particular, bedding, curtains, and carpets are surfaces prone to allergen accumulation [26]. However, conventional vacuum cleaners may resuspend allergens into the air, and therefore tools equipped with HEPA filters are recommended.

Behavioral measures such as restricting animal access to bedrooms, handwashing after contact, and selected cleaning or grooming practices may contribute to lower exposure, but generally provide partial rather than complete reduction. Recommendations should be tailored to the individual and should not imply that lowering a measured environmental allergen concentration guarantees symptom control.

### 9.3. Standard Pharmacotherapy

Standard pharmacotherapy remains central to the treatment of symptomatic cat and dog allergy and should be selected according to the affected organ system and disease severity. Options include second-generation H1 antihistamines for allergic rhinitis and conjunctival symptoms and intranasal corticosteroids for persistent allergic rhinitis [58]. For patients with asthma, treatment should follow current asthma guidelines and include inhaled corticosteroid (ICS)-containing therapy; treatment with as-needed short-acting β2-agonist (SABA) alone is not recommended [59]. These medications treat the patient’s inflammatory and symptomatic disease but do not eliminate sensitization or environmental allergen exposure; therefore, pharmacotherapy should be integrated with exposure management and, in selected patients, allergen immunotherapy or biologic treatment.

### 9.4. Allergen Immunotherapy

Allergen immunotherapy (AIT) aims to induce durable immunological tolerance through repeated administration of clinically relevant allergen preparations [28,32]. Its use in cat and dog allergy requires careful patient selection, confirmation that the animal allergen is clinically relevant, and attention to the composition and standardization of the extract.

AIT is associated with changes in T-cell regulation, effector-cell responsiveness, allergen-specific IgE, and blocking antibodies such as IgG4. Increased IgG4 can accompany successful AIT but should not be used as an individual surrogate of clinical efficacy. Clinical benefit must be evaluated by validated symptom, medication, and disease-control outcomes.

Evidence should be considered separately for cat and dog allergen immunotherapy. Controlled studies using standardized cat extracts have demonstrated benefit in selected cat-allergic patients, including reduced symptoms during cat exposure and decreased cat-specific bronchial responsiveness [60,61]. The evidence base for dog AIT is less convincing because dog extracts are molecularly heterogeneous, standardization is challenging, and the number of high-quality randomized controlled trials is limited [4,53]. Consequently, efficacy should not be generalized from cat AIT to dog AIT or from better-established indications such as house dust mite or pollen allergy.

Despite its benefits, AIT requires long-term treatment, typically for 3–5 years, and is also limited by cost and patient compliance [29,62]. Some patients may experience local or systemic adverse reactions during treatment, and rare cases of anaphylaxis have also been reported [28]. Various approaches including sublingual immunotherapy, in addition to conventional subcutaneous immunotherapy, have been developed, and research aimed at improving patient convenience and safety continues.

### 9.5. Novel Therapeutic Strategies

Source-directed allergen-neutralization strategies are of interest because they aim to reduce exposure without removing the animal. Diets containing egg-derived anti-Fel d 1 IgY have been shown to reduce immunologically active Fel d 1 in cat saliva or on hair [63]. However, reduction in active Fel d 1 is an allergen-exposure outcome and should not be equated with proven clinical benefit in allergic people; evidence demonstrating clinically meaningful improvement in patient symptoms remains limited.

A clinically distinct passive immunotherapy strategy uses two fully human Fel d 1-neutralizing IgG4 monoclonal antibodies, REGN1908 and REGN1909, administered directly to the allergic patient rather than reducing allergen production at the animal source. In a randomized, double-blind, placebo-controlled trial, a single dose of REGN1908–1909 reduced nasal symptoms after controlled cat-allergen challenge and inhibited IgE-mediated and type 2 inflammatory responses [64]. A subsequent randomized environmental-exposure-unit study in cat-allergic patients with mild asthma found that a single 600 mg dose significantly attenuated allergen-induced FEV1 reductions and early asthmatic responses for up to 85 days [65]. These findings provide proof of concept for passive Fel d 1 neutralization in humans, but this patient-directed monoclonal-antibody approach is mechanistically and clinically distinct from source-directed anti-Fel d 1 IgY diets.

Recombinant or modified Fel d 1 molecules and peptide-based approaches are being investigated as strategies to improve the specificity or tolerability of immunotherapy [66]. Biologic therapies such as anti-IgE therapy are established for defined clinical indications, including selected patients with severe allergic asthma, but should not be presented as general treatments for cat or dog sensitization [67].

Other approaches, including peptide-based immunotherapy and allergen-neutralizing strategies, remain investigational [63,66]. Their clinical value requires controlled trials demonstrating patient-centered outcomes as distinct from laboratory evidence of immunological change or allergen reduction.

Animal allergies cannot be adequately managed using a single approach alone, and multifaceted strategies integrating allergen avoidance, environmental control, immunotherapy, and emerging therapeutic approaches may be required. Personalized management strategies that integrate molecular sensitization profiles with clinical manifestations, exposure history, and disease severity may support more individualized clinical decision-making. For example, molecular diagnostic approaches such as CRD may refine molecular sensitization profiles and provide complementary information for individualized clinical decision-making when interpreted together with exposure history and clinical phenotype [3].

## 10. Conclusions

Human allergy to cats and dogs is clinically important, but population-level trends in pet ownership, urbanization, or indoor living should not be interpreted as direct proof of a corresponding increase in allergic disease. These societal factors can modify opportunities for exposure, whereas sensitization and symptomatic allergy arise from interactions among allergen dose and timing, molecular sensitization, host susceptibility, barrier and immune responses, and disease phenotype.

Cat- and dog-derived allergenic proteins can persist in dust and be transported into environments without resident animals, but environmental detection does not necessarily indicate a clinically relevant exposure for every individual. Similarly, polysensitization may be associated with more complex phenotypes in some cohorts, yet the number of positive IgE responses alone does not determine disease severity. Clinical interpretation requires molecular sensitization profiles to be linked with symptoms, exposure patterns, and individual immune and airway characteristics.

Molecular allergology has improved the characterization of cat and dog sensitization and can help distinguish species-associated marker sensitization from patterns compatible with cross-reactivity. CRD is therefore a valuable diagnostic support tool, but it does not independently establish the causal allergen or dictate treatment. Where uncertainty persists, exposure–response concordance and, in selected specialist settings, provocation or functional testing can provide additional evidence.

Management is best framed as a layered strategy that combines clinically appropriate pharmacotherapy with feasible exposure reduction and, for selected patients, allergen immunotherapy or disease-specific biologic treatment. Evidence for cat AIT is more established than for dog AIT, and emerging source-control approaches such as anti-Fel d 1 IgY should be evaluated separately for allergen reduction and for patient-centered clinical benefit. Future research should prioritize standardized molecular exposure assessment, prospective linkage of component profiles to clinical phenotypes, high-quality dog-AIT trials, and intervention studies that measure both environmental allergen reduction and meaningful symptom outcomes.

A precision approach to cat and dog allergy therefore depends less on any single test or exposure measurement than on the integration of molecular sensitization, exposure–response relationships, inflammatory phenotype, and patient-centered outcomes.

## Figures and Tables

**Table 1:** WHO/IUIS-recognized cat and dog allergen components, representative sensitization frequencies, and interpretive considerations.

Animal	Allergen	Protein Family	Reported Sensitization Frequency *
Cat	Fel d 1	Secretoglobin (uteroglobin)	94–96% among subjects with positive cat-extract IgE [12]
Cat	Fel d 2	Serum albumin	14–23% in two cat-allergic cohorts [13]
Cat	Fel d 3	Cystatin-A	60–90% IgE reactivity by plaque immunoassay in sera from cat-allergic patients; this estimate should not be interpreted as population prevalence [14]
Cat	Fel d 4	Lipocalin	63% of cat-allergic subjects [15]
Cat	Fel d 5	Immunoglobulin A	38% IgE reactivity to cat IgA, with substantial carbohydrate-dependent cross-reactivity [16]
Cat	Fel d 6	Immunoglobulin M	26% of cat-sensitized children [17]
Cat	Fel d 7	Lipocalin	38% of cat-allergic sera [18]
Cat	Fel d 8	Latherin-like protein	19% of cat-allergic sera [18]
Dog	Can f 1	Lipocalin	75% of dog-allergic subjects [5]
Dog	Can f 2	Lipocalin	25% of dog-allergic subjects [5]
Dog	Can f 3	Serum albumin	Up to 35% of dog-allergic patients [19]
Dog	Can f 4	Lipocalin	35% of dog-allergic subjects [20]
Dog	Can f 5	Prostatic kallikrein/arginine esterase	70% of dog-allergic subjects [21]
Dog	Can f 6	Lipocalin	38% of dog-sensitized subjects [22]
Dog	Can f 7	NPC2 (MD-2-related lipid-recognition family)	10–20% of dog-allergic individuals [23]
Dog	Can f 8	Cystatin	~12% of pet dander–sensitized patients [24]

* Sensitization frequencies may vary according to study population and assay methodology.

**Table 2:** Diagnostic approaches for pet allergy.

Category	Method	Role	Strengths	Key Limitations/Interpretation
Conventional	Skin prick test	Documents IgE sensitization using allergen extract in skin	Rapid, widely available, clinically familiar	Positive result shows sensitization, not clinical causality; extract composition and technique vary
Conventional	Specific IgE assay	Measures serum IgE to cat/dog extract	Quantitative, reproducible, useful when SPT is unsuitable	Does not define the causal molecular component; clinical concordance is required
Molecular	Component-resolved diagnosis	Measures IgE to individual allergen molecules	Refines molecular sensitization profile and can suggest marker vs. cross-reactive patterns	Does not independently establish clinical allergy; panel availability and interpretation vary
Molecular	Multiplex assay	Measures IgE to many components simultaneously	Broad molecular profiling from a small sample	Complex interpretation; analytical positivity may not equal clinical relevance

**Table 3:** Management strategies for pet allergy.

Category	Strategy	Description	Advantages	Limitations
Avoidance	Allergen avoidance	Removal or reduction in pet exposure	Can reduce clinically relevant exposure when feasible	Often impractical; exposure reduction does not guarantee symptom elimination
Environmental control	Air filtration (high-efficiency particulate air), cleaning	Reduction in airborne and surface allergens	Can lower measured airborne or reservoir allergen	Clinical benefit is variable and may not parallel allergen reduction
Environmental control	Behavioral modification	Restrict pet access, hygiene practices	Easy to implement, low cost	Partial reduction only
Immunotherapy	Allergen immunotherapy	Repeated allergen exposure to induce tolerance (subcutaneous immunotherapy/sublingual immunotherapy)	Potential disease-modifying effect in selected patients	Evidence stronger for cat than dog AIT; long duration and extract-standardization issues
Biologics	Anti-IgE/anti-IL-4/13 therapy	Targeted immune modulation (e.g., omalizumab)	Effective for defined severe/type-2 disease indications	Not a general treatment for pet sensitization; disease-specific eligibility and cost
Emerging strategies	Allergen neutralization	Fel d 1-targeting IgY, novel interventions	Can reduce active Fel d 1 or target allergen-specific pathways	Allergen reduction must be distinguished from proven clinical benefit
Pharmacotherapy	Antihistamines, intranasal corticosteroids, inhaled controller therapy, ocular treatments	Guideline-based symptom and inflammation control according to organ system	Established first-line role in rhinitis/asthma management	Does not remove sensitization or environmental exposure

## Data Availability

No new data were created or analyzed in this study. Data sharing is not applicable to this article.
